# The use of mixture model theory in CFD for the chemical reaction between CO_2_ and soda lime in closed circuit rebreather scrubbers

**DOI:** 10.1186/2193-1801-2-578

**Published:** 2013-10-30

**Authors:** Shona Cunningham, Aoife Burke, Ger Kelly

**Affiliations:** Department of Mechanical, Medical Engineering Design and Innovation Centre (MEDIC), Biomedical and Manufacturing Engineering, Cork Institute of Technology, Cork, Ireland; Department of Mechanical, Biomedical and Manufacturing Engineering, Cork Institute of Technology, Cork, Ireland

**Keywords:** SCUBA, Closed Circuit Rebreathers, Modelling CO_2_, Mixture model theory

## Abstract

A mixture model simulation is presented by modeling the axial scrubber in a Closed Circuit Rebreather (CCR). The mixture model is a good substitute for the full Eulerian multiphase model because the interphase laws are unknown in this case. Analysis of mesh size, mesh type and inflation are made to independently characterize their accuracy by means of convergence before further comparisons with experimental data. The importance of mesh refinement is demonstrated near the wall with satisfactory results seen on the near grid wall of the boundary where a finer mesh is utilized. The contribution of inflation and grid independence to the accuracy of the model is presented in the results section.

## Introduction

**Figure 1:**
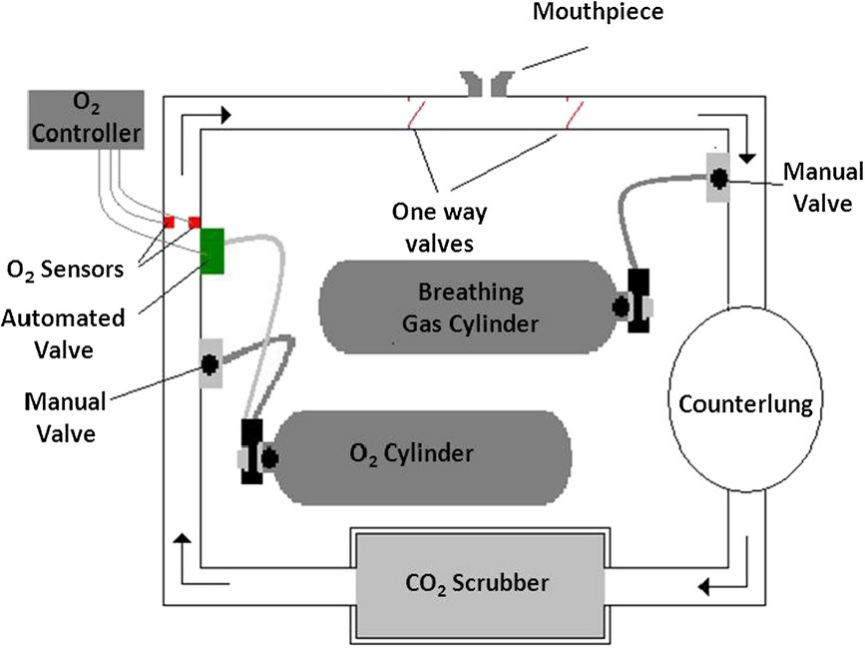
**Closed Circuit Rebreather schematic.** A complete automated system is shown for gas addition. Lines denote direction of gas flow.

A CCR is a class of Self Contained Underwater Breathing Apparatus (SCUBA) used by the diving community, miners and fire fighters. The term rebreather and how one imagines it falls into the common domain of open-circuit SCUBA where exhausted gas or breath is expelled in the form of bubbles from the mouthpiece (Klos 2008). Inefficiency occurs in the open-circuit system as significant amounts of oxygen gas are wasted as the diver exhales periodically. A typical schematic of CCR operation is shown in Figure 1 with CO_2_ laden exhaled breath leaving the mouthpiece of the diver in which it is absorbed by a chemical compound, generally soda lime, lithium hydroxide or Baralyme (Wang 1975) in the scrubber, the latter not seen in rebreathers for over 35 years. The development of a transient model simulating the chemical reactions which occur between CO_2_ and soda lime would greatly enhance the fundamental understanding of these systems and may lead to an optimum design in these systems.

Prior to Clarke (Clarke 2001), the kinetics of CO_2_ absorption in scrubbers was poorly understood. A stochastic method simulating a bed containing a minimum of 200,000 volume elements or cells was employed and within each cell, the temperature and quantity of CO_2_ stored for each time increment was defined. The model was constrained by physics, the chemical absorption within each cell with its resulting temperature are probabilistic as opposed to the chemical properties of the absorbent or CO_2_ which means mass and heat transfer are also determined stochastically. The outputs of the analysis were comprised of a model simulating CO_2_ absorption and thermal fluctuations however the model is only applied to axial design based scrubbers.

Dongsik, Fumin and West (Dongsik et al. 2011) analyzed imperfect CO_2_ removal mechanisms of CO_2_ scrubbers. Their work introduced a stochastic model for three CO_2_ related rebreather faults: (i) CO_2_ bypass, (ii) scrubber exhaustion and (iii) scrubber breakthrough. The authors proposed a stochastic process driven by a Poisson counter to characterize the concept of CO_2_ channeling and the three stated rebreather faults. In probability theory, a Poisson process is a stochastic process which counts the number of events and the time that these events occur in a given time interval. The aforementioned work also advances the understanding of breathing dynamics associated with CCRs and how to maximize performance in terms of breathing and peak to peak pressure. This model is constrained as it does not model the chemical reactions occurring within the scrubber.

Nuckols and Sexton (Sexton & Nuckols 1983) presented a model based on a concept of nodes placed in a flow circuit where mass might conceivably accumulate. The model is based on the concentration of mass, ∆*m* which can be expressed in terms of density of gas in the node and the node’s volume as,
1

where ∆V is the change in volume during a time increment ∂ and ∆*ρ* is the change in density during time increment ∂.

**Figure 2:**
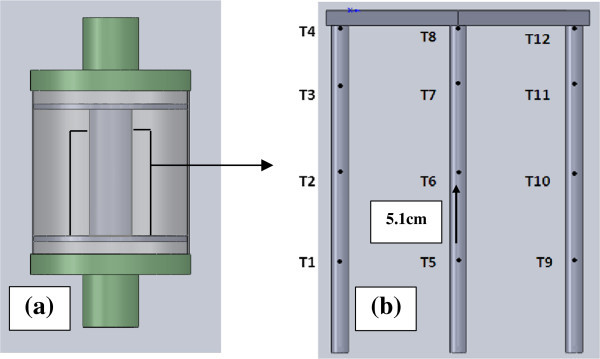
**Test rig set-up. (a)** Axial Scrubber and **(b)** Schematic of rig for thermocouple positions in the axial scrubber.

A review of the literature suggests that the current state of the art in CCR modeling excludes the chemical reactions themselves. This work aims to characterize the chemical reactions by implementing a laminar flow mixture model. To achieve a simulation of the chemical reaction, the properties of CO_2_ and soda lime are analyzed. The chemical reaction between soda lime and CO_2_ is an exothermic reaction and generates humidity (Nuckols et al. 1985). A liquid and gas phase process occurring within the reaction is identified which must be taken into account when modeling the reaction (Olutoye & Eterigho 2005). The absorption of CO_2_ by sodium hydroxide is accompanied by a chemical reaction to form sodium carbonate as a by-product (Olutoye & Eterigho 2005). To validate the proposed methodology of the mixture model theory in this paper, a detailed study of the dependence of the results on mesh density, convergence criteria, mesh type, inflation, aspect ratio and skewness is presented. The simulated temperature rise during the exothermic reaction is benchmarked against measurements taken from an experimental design. The transient temperature rise throughout the scrubber canister is analogous to a moving temperature front. The CO_2_ gas is absorbed by the first layer of soda lime granules and once the soda lime reaches its limit to absorb CO_2_, the reaction continues to the next layer. In order to compare the temperature results directly to the CFD results a thermocouple rig illustrated in Figure 2 was placed into the axial canister.

The average of (T1, T5, T9), (T2, T6, T10), (T3, T7, T11) and (T4, T8, T12) are taken to obtain a temperature for that layer of soda lime granules for any given time.

### Proposed modeling approach

The flow can be classified as laminar in CCR scrubbers (Nuckols et al. 1985). Laminar flow is a type of gas or fluid flow in which the fluid travels smoothly or in regular paths, in contrast to turbulent flow in which the fluid undergoes irregular fluctuations and mixing. The modeling approach uses a laminar model governed by the unsteady Navier–Stokes equations which describe the processes of momentum, heat and mass transfer. These partial differential equations were derived in the early 19^th^ century and have no known general analytical solution but can be discretized and solved numerically. The derivation of the Navier–Stokes equations begins with an application of Newton’s second law: conservation of momentum (often alongside mass and energy conservation) being written for an arbitrary portion of the fluid. In an inertial frame of reference, the general form of the equations of fluid motion is:
2

where v is the flow velocity, *ρ* is the fluid density, *p* is the pressure, *T* is the (deviatoric) stress tensor, and f represents body forces (per unit volume) acting on the fluid (Manninen et al. 1996). There are a number of different solution methods that are used in commercial CFD codes. The method used in Ansys CFX 13.0 is the finite volume technique. This technique divides the area of interest into smaller sub-regions, called control volumes. The equations are discretized and solved iteratively for each control volume (ANSYS CFX 2006). The result gives an approximation of the value of each variable at specific points throughout the domain. This describes a full picture of the behaviour of the flow. Mean particle Reynolds number is used to verify the decision of the laminar model, a calculation is carried out where; *ρ* is the gas density of CO_2_,  is the superficial velocity, *e* is the particle diameter of the soda lime and *μ*. is the dynamic viscosity of CO_2_ in Eq. 3 (Rhodes 1989).
3

Taking the density of CO_2_ gas as 1.87 kg/m^3^, the Re number can be shown to be 0.189 for a particle diameter of 0.0011 m, velocity of 0.0126 m/s and a dynamic viscosity of 0.0001372 kg/ms. The laminar conditions apply up to Re=10 (Rhodes 1989). Within the laminar model, mixture model theory and Henry’s law were applied in the simulation.

### Mixture model theory

The mixture model is a type of multiphase system fined as a mixture of the phases of solid, liquid and gas. Multi-phase flow phenomena are typically dominated by one phase and another non-dominating phase e.g. dust in air (Manninen et al. 1996. However in the case presented in this paper the secondary phase or non-dominating phase cannot be neglected due to the influence on the fluid dynamic behavior of the mixture. The model contains an air|liquid pairing where the air is the inlet gas and the liquid is a reacting component. The decision of modeling threaction as a liquid-particle mixture is based on literature which states absorption as “*the removal of one or more selected components from a mixture of gases by absorption into a suitable liquid is the second major operation of chemical engineering that is based on interface mass transfer controlled largely by rates of diffusion”*(Sinnott 1996). Gas absorption occurs when a mixture of gas comes into contact with a liquid for the purpose of dissolving one or more components of the gas mixture in the liquid. Thus the absorption of CO_2_ occurs with the NaOH component of soda lime in the liquid phase (Physical and Engineering Data 1978).

The soda lime granules are modeled as a block porous media where the inlet gas contains the CO_2_ required for the chemical reaction. Strong coupling between the phases of CO_2_ and soda lime is necessary in this model for liquid-particle mixtures (Ishii 1975). The motions of individual components are treated in terms of diffusion through the mixture. A homogeneous flow within the mixture model is applicable when the phases are strongly coupled in drag dominated flow and their velocities equalize over short spatial length scales (Bowen 1976; Joseph et al. 1990; Johnson et al. 1991). All phases are assumed to move at the same velocity. The volume fraction of the soda lime granule is assigned in the porosity media. The continuity equation for the mixture is denoted in Eq. 6,
456

where *α*_*K*_ the volume fraction of the phase *K*, *ρ*_*K*_ is the average material density, *u*_*K*_ is the local instant velocity of phase *K* and *ρ*_*m*_ is the local density of the mixture (Ishii 1975).

### Henry’s Law

Enry’s law is employed to describe the equilibrium condition for absorption/dissolution of a dissolved gas from a liquid into a gaseous mixture. Henry’s law states the amount of gas dissolved at equilibrium in a given quantity of a liquid is proportional to the pressure of the gas in contact with the liquid. Henry’s law is applied, assuming CO_2_ is a simple gas to describe the equilibrium between vapour and liquid. This law has been employed previously (Farajzadeh et al. 2009; Farajzadeh et al. 2007) in the sequestration/capture of CO_2_. The application of Henry’s law to calculate CO_2_ yields a value 29.41 Latm/mol from Eq. 7,
7

where *p* is the partial pressure of the solute in the gas above the solution, c is the concentration of the solute and k_H_ is a constant.

### The three step reaction

The reaction between soda lime and CO_2_ is a three step exothermic reaction producing water vapour. Eq. 8 describes how gaseous CO_2_ dissolves in water which is the first of the three step reaction (Reid et al. 1987).
8

The second equation is stated below in Eq. 9. This shows that the strong base in this case NaOH is not consumed but acts as a catalyst in the reaction. This bicarbonate is formed due to the high pH.
9

The final step shown in Eq. 10 deals with the production of calcium carbonates,
10

Combining the preceding three reaction steps result in an overall reaction in Eq. 11 where the heat release is 16.4 Kcal/mol of CO_2_ (W.R. Grace and Co. 1986).
11

Water is required to initiate the CO_2_ absorption (Eq. 8). However water is a by-product of the chemical reaction that takes place within the canister (Eq. 9). If the incoming gas stream is saturated with water vapour, an excess of water vapour will remain in the canister. This excess water coats the soda lime granules and cause blockages in the pores. The CO_2_ does not absorb as efficiently and this may also cause caustic vapour in the loop which could burn the diver’s throat. Conversely if the incoming gas stream is too dry, the commencement of the reaction may be limited or the absorbent bed may be too dried out, thereby preventing absorption. Moisture levels of the incoming gas stream should be maintained above 70% RH when using soda lime.

### Boundary conditions and assumptions

The simulation was performed using the CFD software program Ansys 13.0 CFX and the following assumptions are made for the model; (i) the CO_2_ is absorbed fully without loss until breakthrough, where breakthrough is defined as the time until the canister effluent/CO_2_ passes through the soda lime granules unscrubbed, (ii) the CO_2_ gas is uniformly distributed throughout and (iii) a constant CO_2_ injection rate is employed. The axial scrubber is analyzed using CFX and is comprised of a packed bed of soda lime granules modeled as a porous media in which exhaled breath (5% CO_2_, 16% O_2_, 78% N_2_) and traces of water vapour (William et al. 2009) is passed through the scrubber at an inlet velocity of 0.0126m/s. When the composition of exhaled breath passes through this porous media, a series of chemical reactions take place absorbing the CO_2_ and producing water vapour and heat. The inlet gas has a static temperature of 25°C initially at atmospheric conditions.

The continuous governing equations are converted into algebraic equations by using the finite volume method. These subdomains or boxes allow the analysis of flow in each box individually and then these fluid portions can be collated to yield a complete picture of fluid flow in the entire domain of the scrubber.

### Modelling the geometry

**Figure 3:**
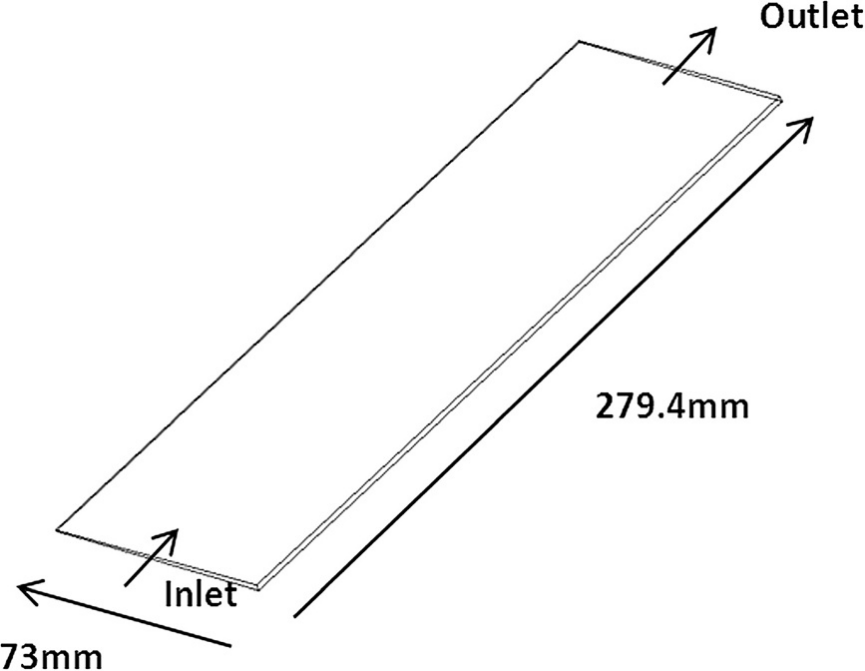
**Schematic of the model axial scrubber slice.**

The geometry illustrated in Figure 3 is a 1° degree segment due to the assumed homogenous flow to represent a full 360° degree cylindrical canister, length 279.4 mm, width 73 mm and spherical particle diameter of the soda lime grade 1.1 mm. The segment encompasses the porous medium. It was decided to model the porous medium as a “block” rather than attempting to model the flow through/around each individual granule. The porosity and particle diameter is specified as part of the physics definition in CFX. The flow domain consists of gas travelling axially from the inlet to the outlet as illustrated in Figure 3.

### Mesh type

**Figure 4:**
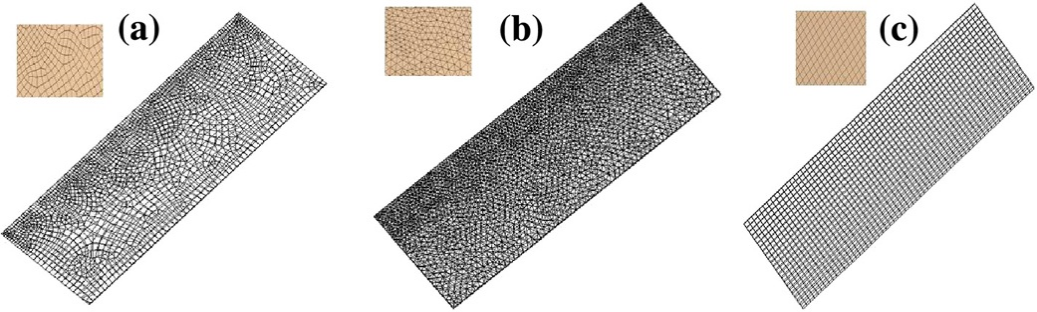
**Schematics of the axial scrubber segments meshed (a) Hex, (b) Tet and (c) Swept/Auto mesh.**

Once a model of the flow geometry is created, it then needs to be meshed. The mesh defines the locations at which the flow solution is computed. In general, meshing the segment is usually a choice between a structured grid, unstructured grid or mixture of both. Typically a structured mesh is comprised of hex elements that follow a uniform pattern. An unstructured mesh does not follow a uniform pattern and is usually comprised of tet elements. Three different mesh types were created for the axial scrubber segment (i) hexahedron mesh (hex mesh) with square/rectangular elements, (ii) tetrahedron mesh (tet mesh) with triangular elements and (iii) swept/automatic mesh as randomly generated by the computer. Initially each mesh was generated automatically in order to analyze the comparison in the convergence of the iterative solution used in CFX. In order to obtain convergence criteria for the models, it is necessary to check the residuals, relative solution changes and other indicators to make sure the iterations converge. A minimum convergence criteria of 1e^-4^ is given as a prescribed tolerance (Pordal 2006a). The accuracy of the solution requires a balance between the number of elements used in the grid, the type of mesh and the time taken for the iterative computation of the model. The automatic hex mesh illustrated in Figure 4 (i) generated an unstructured mix of hex and tet elements with their characteristics given in Table 1.

**Table 1:** **Table of initial mesh details**

	Hexahedron	Tetrahedron	Sweep/Automatic
**Nodes**	4129	4815	2852
**Elements**	5697	13678	1342
**Duration of computation**	10 h 52 mins	17 h 54 mins	4 h 18 mins

In order to initiate this comparison the different types of mesh, the number of elements and the duration of computation are given in Table 1. The results varied in computational duration with the tet mesh the most computationally expensive.

**Figure 5:**
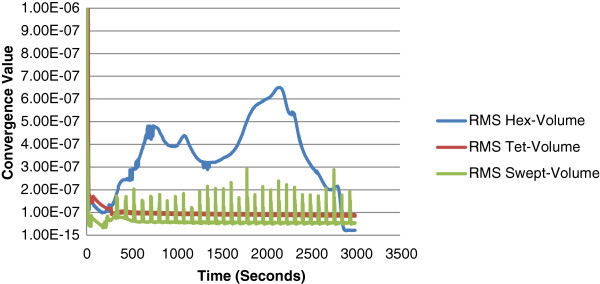
**Convergence criteria graph for the initial generated meshes.**

The elements were generated automatically for each discretization scheme and though the number of elements varies between each type, the tet mesh was the only one to converge as illustrated in Figure 5. The swept mesh was closer to convergence even though it was oscillatory in nature and the hex mesh showed little signs of convergence. The hex mesh would ideally contain all quad elements however since the automatic mesh generated some tri elements, this makes the grid unstructured which will affect the accuracy of the solution. The results highlight the lack of convergence in the unstructured hex mesh in comparison to the swept mesh which is completely structured and contains the least amount of elements.

**Figure 6:**
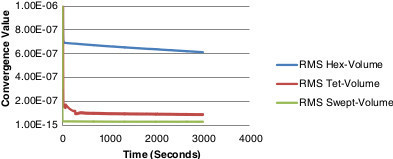
**Convergence criteria graph for the modified meshes.**

In order to attain an accurate comparison between the meshes, the same number of elements should be used. This would involve increasing the number of elements in both the hex and swept meshes to obtain convergence. Table 2 outlines the modified mesh details. When the elements are increased in both the hex and swept mesh, convergence occurs in both as illustrated in Figure 6. The results show the swept mesh is better converged than the hex and tet meshes respectively as it reaches a convergence criterion of 1e^-15^ and is computationally less expensive in terms of the time to reach convergence. A structured grid was therefore chosen as the optimum mesh.

**Table 2:** **Table of modified mesh details**

	Hexahedron	Tetrahedron	Sweep/Automatic
**Nodes**	15050	4815	15686
**Elements**	12735	13678	13420
**Duration of computation**	19 h 2 3mins	17 h 54 mins	16 h 48 mins

**Figure 7:**
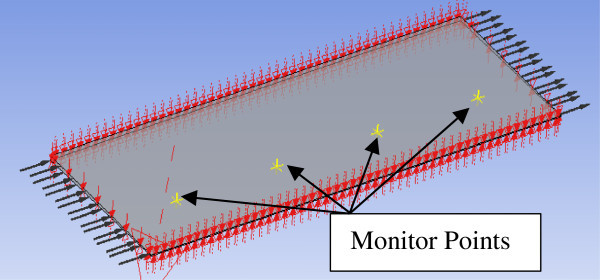
**Illustration of the monitor points placed in the bed length of the axial scrubber denoted by yellow markers.**

The analysis of convergence can reliably be undertaken to provide an element of confidence independently using the CFD model before comparative work with the experimental results. The temperature rise generated from the exothermic reaction of the simulations for the different types of mesh are compared to the experimental data obtained from the thermocouples placed on the constructed rig as discussed earlier. The average of (T1, T5, T9) and (T2, T6, T10) are referred to as 'Exp1’ and 'Exp2’ respectively for the comparative work. Monitor points were placed at the same geometrical positions along the bed length in the CFD model to obtain a direct comparison as shown in Figure 7.

**Figure 8:**
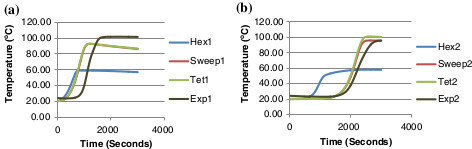
**Mesh type comparison against experimental data (a) comparison of point 1 (b) comparison of point 2.**

**Figure 9:**
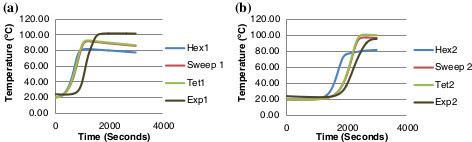
**Converged mesh type comparison against experimental data (a) comparison of point 1 (b) comparison of point 2.**

Hex 1 denotes the first monitor point/thermocouple e.g. 'Hex1’ and the number 2 signifies the second monitor point/thermocouple e.g. 'Hex2’. Figure 8(a) and (b) graphs the temperature results of the initial meshes generated against the experimental data and Figure 9(a) and (b) graphs the temperature results for the converged meshes against the experimental data. The experimental data is obtained from the thermocouples as the heat generated from the exothermic reaction moves along the bed length of the scrubber. As concluded from the convergence analysis, the hex mesh is the least accurate. The hex mesh values in Figure 9(a) and (b) display a greater level of accuracy relative to the previous graph; the values are still inaccurate by 20°C compared to the experimental data.

The computational run time using this hex mesh was recorded at 19 h 23 mins. This mesh is the least accurate and the most time consuming. The tet and swept mesh both exhibit convergence and accurate similar values in comparison with the experimental data. The swept mesh is computationally less time expensive and structured so it was deemed the best system.

There is also a strong interaction between modeling errors and the time and space resolution of the grid. The quality of the mesh can be determined by many different factors (i) mesh type, (ii) convergence criteria, (iii) inflation, (iv) aspect ratio and (v) skewness of the mesh.

### Inflation test

**Figure 10:**
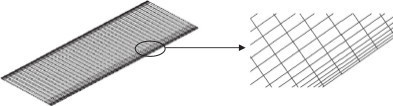
**Schematic of inflation on the walls of the model.**

The advantage of employing a structured mesh is shown through the examples of convergence, computation time and comparison with the experimental data in the previous section. Inflation is another parameter which characterizes the quality of the mesh. Inflation is where a finer resolution grid is placed on/near the walls to analyze the boundary layer of the model without subjecting the whole mesh to the chosen resolution of the inflation layers illustrated in Figure 10. The resolution of the inflated layers is important as the results obtained at the walls of the laminar model presented may affect the accuracy/convergence of the model. Therefore the parameter of inflation will be incorporated and independently tested.

**Figure 11:**
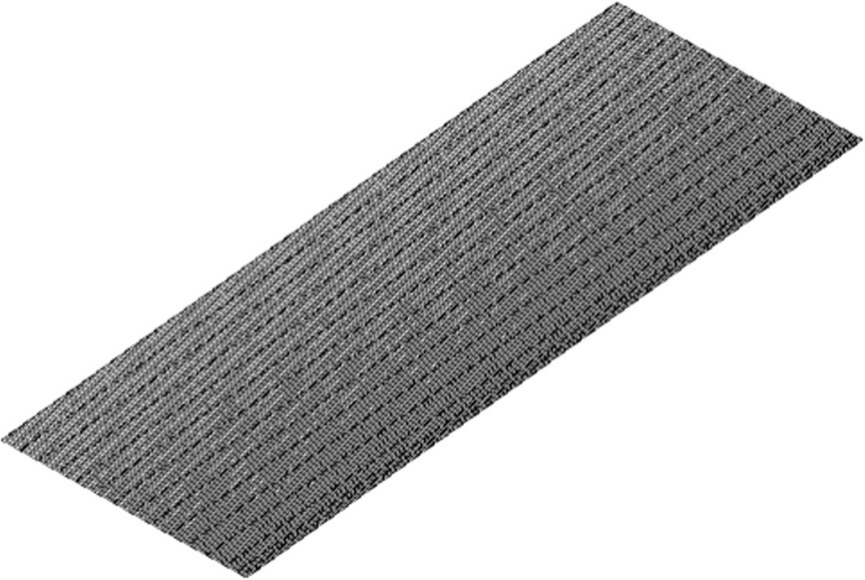
**Schematic of axial scrubber segment with an edge sizing of 400.**

A structured mesh was developed using an edge-sizing on the bed length of the axial model. The edge sizing allows the control of the grid size along the bed wall. The edge-sizing was initially set at 400 as illustrated in Figure 11.

**Figure 12:**
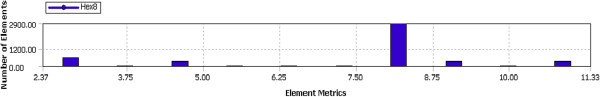
**Aspect ratio chart for a quad grid with an edge length sizing of 400.**

One of the tools used in determining the quality of the mesh grid is the aspect ratio i.e. the ratio of the width of the cell to the height of the cell. For a good/acceptable mesh the maximum aspect ratio value should approximately be 10 (Pordal 2006b). Figure 12 charts the aspect ratio of the elements with a quad grid and an edge sizing 400. The x-axis gives the particular size of an element against the number of elements on the y-axis. The maximum aspect ratio in this grid is 11.33. This value would classify the mesh as poor quality.

**Figure 13:**
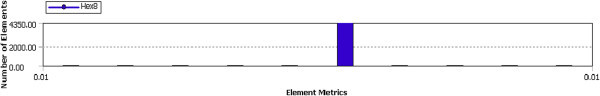
**Skewness for a quad grid with an edge length sizing of 400.**

The other tool used in analyzing mesh quality is the skewness of the element cell. Skewness is defined as how close to ideal (i.e. equilateral or equiangular) a face or cell is. A value of 0 indicates an equilateral cell and a value greater than 0.85 (Baker 2002) is considered unacceptable for a quad cell. For this model the skewness is 5.56e-3 from Figure 13 which defines the quads drawn in the mesh as equiangular.

**Figure 14:**
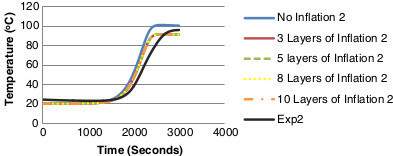
**Temperature comparison of inflation layers against experimental data for monitor/thermocouple point 2.**

In order to refine the mesh, inflation was added to the walls to capture the effect of shear stress on the walls. The level of inflation was varied for the different models and the details are presented in Table 3. In order to compare the effect of inflation against experimental data ('Exp2’ on Figure 14), temperature monitor points were once again used and the results are presented in Figure 14. Initially no inflation is used on the grid and then layers of inflation are increased at the walls.

**Table 3:** **Details of modified inflation layers with a constant quad grid**

	No Inflation	Inflation3	Inflation5	Inflation8	Inflation10
**Convergence**	Near convergence	Near convergence	Near convergence	Near convergence	Converged
**Aspect Ratio**	11.33	6.16	5.042	6.9746	10.978
**Skewness**	5.56e-3	0.6	0.6	0.6	0.6
**Elements**	4350	13200	14800	16000	16800
**Nodes**	9312	26867	30075	32080	34085

Figure 14 illustrates the need for inflation in laminar flow against the walls of the axial scrubber segment as the temperature values particularly in 'No Inflation 2’ are not only too high but there is a greater time lag observed. Due to the slow nature of heat transfer, there is no difference between 3, 5, 8 or 10 inflation layers as the results collapse onto each other in both graphs. It is shown there is a need for inflation against the walls when analyzing heat transfer. Due to the difference in convergence, 10 inflation layers are used for the grid independence test even though it is 0.978 more than a good aspect ratio from Table 3.

### Grid independence test

**Figure 15:**
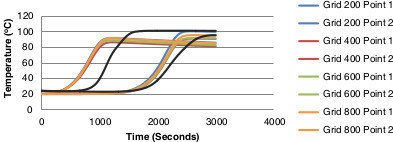
**Temperature comparisons for grid independence test against experimental data.**

Improper grid sizes can contribute to inaccurate results so it is important not only to look at the inflation layers but also the grid size when analyzing the mesh, where the grid is the geometry of the cells/elements used to build the mesh. The grid is increased in edge sizing until convergence is reached and a repeated or acceptable result is achieved from the grids. The details of each grid are displayed in Table 4. The 400 and 600 grids should have the most accurate results as both have converged with acceptable skewness and aspect ratios in comparison to the 200 and 800 layered grids. The accuracy in temperature prediction with the refinement of each grid is noticeable in Figure 15. The improvement to temperature prediction due to a finer grid may be considered unnecessary as the accuracy provided by the 600 grid would be acceptable in many engineering applications. Taking into account computation time for the runs, finer meshes giving similar results are a disadvantage. The 600 grid is therefore decided upon as the favoured/optimum mesh.

**Table 4:** **Details of modified edge-sizing grid with constant inflation layers**

	Grid200Inflation10	Grid400Inflation10	Grid600Inflation10	Grid800Inflation10
**Convergence**	Near convergence	Converged	Converged	Converged
**Aspect Ratio**	10.978	10.978	9.2437	12.3
**Skewness**	0.6	0.6	0.6	0.6
**Elements**	8400	16800	31800	42400
**Nodes**	17085	34085	57095	76095

**Figure 16:**
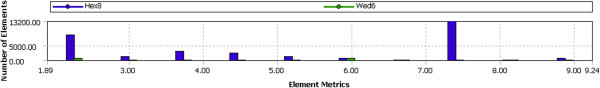
**Aspect ratio chart for the Grid600Inflation10 mesh.**

**Figure 17:**
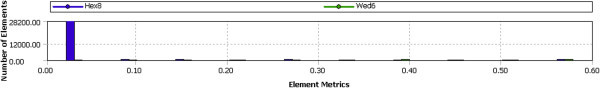
**Skewness chart for the Grid600Inflation10 mesh.**

The aspect ratio and skewness charts are presented below in Figures 16 and 17 for the final mesh of Grid600Inflation10. Table 4 lists the maximum aspect ratio as 9.2437 but Figure 16 shows the majority of cells are approximately 2 or 7.5 which falls well under the ratio of 10 acceptable for the aspect of the cell. Likewise the maximum skewness is 0.6 however the majority of cells have a skewness factor of less than 0.1 which is an indication of an excellent mesh quality.

### Grid convergence study using Richardson extrapolation

To accurately characterize the convergence solution, both Roache (Roache 1994) and Oberkampf and Blottner (Oberkampf & Blottner 1998) encourage the use of at least three grid levels to obtain the error estimation of the convergence of the chosen grid. The convergence study requires a minimum of three grid solutions. To complete this study, first the grid refinement ratio (r) is calculated. The level/ratio from which grid 2 is refined in relation to grid 1 and grid 3 in relation to grid 2 is the grid refinement ratio which in this study is 1.9 as calculated from Eq. 12 (Roache 1994). The grid details are listed in Table 5.
12

**Table 5:** **Details of the grids used for GCI**

	Grid 1	Grid 2	Grid 3
**Nodes**	34085	57095	99567
**Elements**	16800	31800	60420

According to Stern et al. (Stern et al. 2001) the order-of-accuracy can be estimated by using the following equation in Eq. 13. The analysis thus far was undertaken in terms of the temperatures obtained within the scrubber which will once again be used as the parameter to attain the order of accuracy.
13

Where,
14

To evaluate the extrapolated value from these solutions, the convergence conditions of the system must be first determined. The possible convergence conditions are (a) monotonic convergence; 0<R<1, (b) Oscillatory convergence; R<0 and (c) Divergence; R>1 where R is the convergence ratio and it is determined by Eq. 15,
15

Roache developed the Grid Convergence Index (GCI) as a means to uniformly measure the convergence for grid refinement. This may provide the error band of the solution. The GCI for the fine grid solution is defined in Eq. 16. The factor of safety is recommended to be *Fs*=1.25 (Wilcox 1998) for comparisons over three or more grids. Due to the monotonic convergence condition of the results, Eq. 16 is used.
16

Table 6 shows there is a significant reduction presented in the mean average error from the coarser grid to the finer grid. The calculation of *R* gave a result which made the solution monotonic. This is the first indication of good convergence. The GCI solution is calculated at 5% which indicates the chosen grid is an acceptable grid (Roache 1994).

**Table 6:** **Order of accuracy and grid convergence index**

	ϵ _32_	ϵ _21_	R	p	GCI(%)
**Temperature (°C)**_**MAE**_	3.87	0.385	0.099	3.59	5

Subscripts represent the respective grids.

## Discussion

The paper presented a CFD model of a CCR axial scrubber using the mixture model theory to analyze the chemical reaction between soda lime and CO_2_ as an alternative technique to current methods. The mesh density influences the accuracy of the results and thus a benchmark with experimental data of the final mesh was conducted. The first parameter influencing the mesh is the type of mesh chosen for the model. A structured quad mesh was identified as being the optimum as it showed satisfactory convergence and comparison with the experimental data. It was also less computationally expensive to run. The analysis of inflation was conducted on the boundary walls of the model. The level of inflation was varied for the different models and compared against experimental data where it is shown that inflation on the walls will contribute to the accuracy of the model. A grid independence test was carried out to analyze how the fineness or coarseness of the mesh grid influenced the results. The finer the mesh, the more accurate the solution becomes however at significant computational cost. The point at which similar results are seen between two meshes acts as validation in the choice of selecting a 600 grid with 10 inflation layers as the optimum grid. This mesh density is a close match in temperature and produces better results over the time on the x-axis. The aspect ratio and skewness of the cell are also used as a means of validating the mesh independent of experimental results. For the final mesh both the aspect ratio and skewness are acceptable values at 9.24 and 0.6. The relationship between the actual mesh and the experimental data show that the predicted results lag behind the actual experimental profile. This may be attributed to a lag needed in the simulation where the model needs to reach a steady state phase before the temperatures correlate to the experimental data as is seen with the second set of monitor points. The overall trend of the model prediction agrees well with the experimental data. The mesh density; including type, grid size and inflation coupled with the aspect ratio and skewness provide a method of characterizing the mesh. The GCI value of 5% is also an acceptable result. The method presented allows an independent validation of the mesh quality which is further validated with the experimental results.
